# Additional Remarks on Permanent Stricture

**Published:** 1809-05

**Authors:** John Roberton


### *Additional Remarks on Permanent Stricture,*

By John Roberton, M. D.

( Continued from our last. )

WHAT I understand by Stricture in the Urethra *of a permanent nature*, is an evident diminution in its capacity, in consequence of some substance being gradually superadded to that canal; and although, from certain circumstances irritating these parts, even this obstructing body

 should should suffer considerable contraction and relaxation, yet the canal *at no time* can return to its natural dimensions till this body be completely removed.

In every period of the history of the venereal disease, the existence of *permanent* stricture in the urethra has always been, among the better informed part of the medical profession, conceived to be very rare. From time to time, however, men of considerable ingenuity and address have started up, who have found it convenient to maintain a very different doctrine. In short, there is perhaps no disease, the existence of which has been so often in a great measure doubted, and so often conceived frequently to follow one or other stage of gonorrhœa or of gleet, or to be the consequence of the treatment adopted for the removal of these diseases.

Now, although Mr. Hunter informs us, in page 112 of his book on venereal complaints, that permanent strictures are generally attended with gleet, he does not by any means conceive this to be a diagnostic symptom, on which we are, without further investigation, to proceed to the use of caustic. Mr. Home uses less ceremony; for, without any other symptom, or at most that of a very slight spasm only, he introduces the caustic into the urethra. Sometimes in one, at other times in from *fifty* to a *hundred* applications, he arrives at the bladder; the inflammation occasioned by this process, removes the gleet, which might probably have been cured by much easier means. From this success, however, the disease is termed stricture, and is thought of much more frequent occurrence than it really is.

It cannot be too strongly inculcated that permanent strictures, as they are termed, as well as spasmodic ones, are most commonly to be found about the curve of the urethra. This is evidently owing to this part, from its shape, being, in the urethra of every person, most likely to obstruct the progress of the bougie in our attempts to introduce it into the bladder. I think this at least a highly probable conjecture, and I have no hesitation in asserting, that the bougie thus arrested, has in many instances given rise to the supposition of stricture in these parts, and led to a painful and tedious train of the most cruel practice.

In giving an account of the dissection of those said to have died, after the removal by caustic, of permanent stricture in the urethra; there seems to be nothing in the cases which Mr. Home has adduced, to prove that permanent stricture ever existed in the majority of them. The parts said to have borne the marks of stricture might be, and and I suspect many of them were, produced by the cauterizing effects of the application *employed*, not the disease which it was applied to remove. But even if we were to allow that callosities, perhaps to a considerable extent, and stricture of a permanent nature, do sometimes exist in various parts of the urethra, the existence of caruncles and excrescences, formerly as much in *fashion* as permanent strictures now are, seem to me very doubtful. We all know that mucous membranes in general, when ruptured from any cause, such as the lining of the mouth, &c. do not form such appearances; on the contrary, the parts are speedily regenerated, and assume their healthy action. The existence of these caruncles, excrescences, &c. is at length disputed, and the same symptoms imputed to *permanent strictures*. Mr. Home is quite aware of this fact; page 103, he acknowledges, that "if any one will take the trouble to compare Daran's cases with those mentioned in this treatise, he will find that they correspond in the number and situation of the obstructions, and *only differ in the names given them*." A difference exists however; Daran and his cotemporaries removed them without caustic.

That such a disease as permanent stricture in the urethra exists, will not admit of a doubt; but it exists much seldomer than every author who writes on the subject wishes to persuade us.

All membranes, as well as the cellular substance which joins them to the contiguous parts, are often indurated and thickened in consequence of inflammation; this happening to the membrane of the urethra is one form of stricture, which occurs independently of muscular action, and to explain which we stand in no need of hypothesis.

Permanent strictures in the urethra may be produced, though perhaps not very commonly, by severe inflammatory action, however induced, being brought on these parts; but they may, and certainly are, more frequently produced by morbid action in these parts, in consequence of long continued disease.

I should imagine, contrary to Mr. Benjamin Bell's opinion, in page 267 of his first volume, on venereal complaints, that instead of violent inflammatory action most commonly producing that kind of stricture, which feels as if a cord or thread had been bound about the penis, that directly the contrary takes place; that this kind of stricture is produced by long continued and violent spasmodic action of these parts, and that where there is a considerable thickening of some greater extent, it is more likely to

 have have arisen from previous inflammatory action of thes parts.

I may observe that the disease called phymosis, or a contraction of the prepuce, so as to prevent its being drawn back over the glans, gave me a more perfect idea of permanent stricture than any other circumstance; and, previous to my observing this disease from its commencement, I conceived it a strong proof of the probability of permanent stricture being a very common disease. I however found, on carefully attending to its first progress, that its dilatation was easily effected; and when this practice was adopted, the contraction seldom arrived to any degree of severity; but when allowed to take its course, it almost always required to be cut, dilatation seldom being capable of effecting a perfect cure.

Strictures, when they do exist in the urethra, occupy it all round like a thin membranous partition of various breadth; they may, however, be confined to any particular side of the urethra, or they may exist by a thickening of the membrane, or of the parts immediately below it, to the extent sometimes of an inch. Tumours also in the neighbouring parts, from various causes, occasion a greater or less degree of contraction in the urethra, which is certainly permanent while these tumours exist.

In the commencement of any obstruction in the urethra, however slight, or from whatever cause, the diameter of the canal being less or more narrowed, the patient must be sensible of its existence. Mr. Home conceives it difficult to ascertain the existence of obstruction, unless the patient has been attentive to the usual healthy dimensions of his stream of urine.

When we reflect on the diameter of the urethra, we are at once convinced of the impossibility of such a narrow canal being in the smallest degree obstructed, without occasioning a sufficient portion of distress to the patient, to make him at once apply for assistance.

Some degree of spasm may continue for years in the permanently contracted part; this can never be reckoned a consequence of the permanent contraction, but only a degree of the cause still existing, which actually produced the permanent contraction. It seems, however, that writers on the subject of stricture, take quite a different view of the subject: they seem to conceive the spasm in this case to be a sort of symptom, or an attendant on permanent stricture. It is not amiss to observe, that the greater the uniformity of opinion that exists on any any subject, the more tardily does it advance towards perfection; while to difference of opinion we are indebted for many of our most valuable truths.

Mr. Hunter also seems to labour under some confusion, in talking of the spasmodic affection, which sometimes attends what he terms permanent strictures: he observes, in page 134, that "there is sometimes this singular circumstance attending these cases, that when there arises a gonorrhœa, or any other discharge of matter from the urethra, where none was before, or an increase of an old gleet, the passage becomes so free *as to allow the urine to pass as usual*; but these are uncertain, and only temporary reliefs; for whenever the discharge ceases, the spasmodic affection returns. I think it most probable that it is only the spasm that is affected by the discharge, and not the real stricture;" and he was right, for it is ten to one if, under the above circumstances, any such thing as permanent stricture existed; but if there really was such a thing, it must have been very slight, otherwise no alteration but its entire destruction could have allowed *the urine to pass as usual*. At all events, the spasm here must have been the principal disease, and might have been removed by proper applications, *not by caustic.*

Enlargement of the glands of the urethra, which are assuredly most common in scrofulous habits, act while in that state, in some measure, as foreign substances, causing permanent diminution, irritation in the neighbouring parts, and often violent spasmodic action. Under these circumstances, in our present deficiency of knowledge of those means by which glandular swellings are removed, I conceive the very worst cases of stricture, either spasmodic or permanent, to exist. We may alleviate either of these affections; but from what I have stated above, a cure in our present state of knowledge is scarcely to be expected. Glandular swellings in these as well as in other parts, is very seldom a subject on which much is yet to be done.

### Cure of Permanent Stricture.

More than a hundred years ago, caustic applied to the urethra was by no means an uncommon practice. It was soon after, however, laid aside by general consent; yet, during that period, we are not assured that the existence either of permanent or spasmodic contraction in the urethra was more common in consequence of laying aside the caustic; but on the contrary it would seem, from authors

 saying saying little on the subject, to have been less so than in earlier times, when the caustic was employed, than in the present day, when the caustic *cannot be wanted*. Indeed, such is our present rage for that practice, that one can scarcely walk into a country apothecary's shop, but he can tell you wonders (and I have no doubt of it) that he has wrought with the caustic. It is sincerely to be hoped, for the benefit of mankind, that it will soon again be laid aside, except when absolutely necessary, which is very seldom.

The liberties which, upon this subject, Mr. Home has taken with Mr. Hunter's name are greater than we should have expected. That gentleman's opinion respecting the application of caustic for the removal of stricture was, tho' perhaps in some respects erroneous, much more scientific, less common, and, in a great measure, different from the views of Mr. Home on the same subject. Mr. Hunter says, in page 118, of his book on the Venereal Disease, "if the case is such as to admit the end of a small bougie to pass, let it be ever so small, the cure is then in our power." Mr. Hunter thus found that such cases were remediable, but he wanted a knowledge of the means by which he could have effected this purpose. Were, however, Mr. Home as scientific in his views, respecting many points in the animal economy as Mr. Hunter, many who suffer by the effects of the caustic bougie, might have now been in tolerably good health.

The progress of Mr. Home's practice, at least that part of it which he has ushered into the world as a proof of the correctness of his reasoning, and of the *simplicity* and *ease* of such practice, is as follows. His first essay was upon a small scale, and simple enough; from Mr. Hunter's respectability he gained applause, or at least, from that circumstance, he, on that occasion, escaped; he ventured a little farther; proposed something new; but still, for the most part, on such occasions, he took care to shelter himself under the *wing* of Mr. Hunter's well-earned reputation; till at last finding himself pretty nearly in a way that he thought warranted him to speak and act entirely for himself, he boldly asserted, that many of the more important diseases ascribed by Nosologists to other causes, depend entirely on stricture in the urethra.

'I give the surgeons of London, in general, every credit for the superiority of their powers in the performance of operations in general: indeed, I believe that at present they are, with the exception of one, or at most, two individuals, dividuals, superior in that respect to any in the world. But in the discrimination of diseases, I would reverse the statement, for I really think the proportion of them who can do this well is not so great, at least not nearly so great as in other parts of the world, where with their head, and the hands of the surgeons of London combined, the most decided success would attend many operations, which in their divided state are but clumsily managed.

Mr. Sharpe, I may observe, when treating of the subject of strictures in the urethra, informs us, that "at present, it (the caustic) is universally condemned, and has been so almost since Saviard's time. His objections to the use of caustics, were the difficulty and almost impossibility of directing them, so as to eat through all the diseased parts of the urethra, without destroying the sound parts; *the impracticability of preventing the urethra from contracting when it healed, as much, if not more than it was* at the time of applying the escarotic, &c." These objections may with equal justice be always urged against this practice, even where permanent strictures are formed, and far more so when we find it indiscriminately applied, where there is no permanent stricture at all; nor will even the authority of Mr. Home, for the *ease* attending its application, and its *safety*, overturn them. Mr. Sharpe's authority, among men who know how to reason, has always been at least as good as Mr. Home's, and will probably remain longer so.

Mr. Whately has, in defence of the application of caustic, attempted to draw a comparison between its effects and that of the more active medicines used internally; but the comparison is not fair: we can dilute the medicines and use them in any quantity we wish, but we must use the caustic in full quantity, and in its greatest state of strength and activity.

This gentleman, however, has, in his pamphlet on Mr. Home's practice, given a very neat, condensed view of the effect of Mr. Home's bougies, in his treatment by caustic of obstructions in the urethra. His criticisms seem correct, and are certainly those of a gentleman and a man of liberality; yet, even he, in some of the cases subjoined to these criticisms, has used the caustic in pure spasmodic stricture, and consequently subjected the patient to much unnecessary distress. Mr. Whately justly objects to Mr. Home's introducing the caustic bougie into the bladder, from the chances of its carrying along with it, and depositing siting in that viscus, any of the liquified caustic. This is an objection which certainly deserves some consideration.

In a note in page 36 of Mr. Wadd's pamphlet, he informs us, that "to a gentleman, with whom he was well acquainted, it (the caustic) was applied *upwards of fifty times* to a supposed stricture near the neck of the bladder. *When he died, the obstruction was discovered to have arisen from an enlarged prostate gland. The caustic had eaten an inch into the substance of the gland.* Many other instances of a similar kind, " he informs us," might be enumerated. Yet it is passing strange, when on reading Mr. Home's book, we find that the application of the caustic, neither occasions *pain* to the patient, nor *difficulty* to the surgeon.

The communications which I have lately had from many parts of the country respecting various diseases of the generative organs, prove that the caustic bougie had in them not only been unsuccessfully applied, but had created, in some instances, the most incalculably afflicting and incurable misery, to which it was possible to reduce an unfortunate patient. In others, where this practice was not carried so far, I have easily removed their complaints by the instantaneous adoption of other more simple means.

Even allowing all the cases Mr. Home operated for with his caustic bougies, to have been permanent strictures, the frequent recurrence of the affection, always in an aggravated degree, and the necessity there was in some instances of applying the caustic often for a great succession of years, several hundred times in some instances, completely prove that, from such an application, we have no right to expect a radical cure, except in the very slightest cases, and that only when the contracting power has quite abated.

Were no other mischief to follow the application of caustic to the urethra but the *excessive* hemorrhage, which is confessed even by authors who are advocates for this barbarous practice, to be a very frequent occurrence, that alone would deter any one from its constant use. These authors, however, talk with much coolness, when even six or eight pounds of blood have been discharged in this way. Various cases are related by them of the *ease* and *safety* of such bleeding. Mr. Wadd, however, takes another view of the subject. "In one case," says he, "after the eighth application of the caustic, on withdrawing the bougie, I was instantly covered with blood, which came out, with a jet, nearly equal to the flow of urine. I must confess, whatever those accustomed to such accidents may think of it, it, that I was greatly alarmed; and as it happened in my own house, it was the more embarrassing: pressure and cold applications were used in vain; and it was some hours before it became sufficiently moderated, to allow the patient to be carried home in a sedan chair. The bleeding continued, at intervals, for several days; and it was *five months* before the patient, who was a foreman in a manufactory, had recovered his strength sufficiently to resume his station.

However far," Mr. Wadd remarks, " habit may teach a surgeon to regard these circumstances with indifference, he will not so easily succeed in making the patient, or his friends, believe there is no danger, and that extreme debility is a matter of no consequence."

Strictures, however, of a permanent nature, can only be removed by the various modes recommended by authors for their entire destruction; such as the various ways of applying caustic substances, and even some may probably require incision.

Mr. Hunter conceived the caustic properly applied only under the following circumstances. " First, Where the stricture is so tight, as not to admit the smallest bougie to pass. Secondly, Where the orifice in the stricture is not in a line with the urethra. Thirdly, Where the passage has been obliterated by disease, and the urine passes by fistulæ in perinæo." Had Mr. Home followed Mr. Hunter in these plans of practice, and not taken him along with him, as he always does, merely to support him in his difficulties, he would have prevented many an unnecessary scene of misery to several unfortunate individuals.

I have found, where *permanent* stricture *actually existed*, and where it was absolutely necessary to apply some substance for its removal, the mode of applying either the lunar caustic or the *kali purum*, as recommended by Mr. Whately, preferable to any other method of applying such substances to these parts; at least, to any with which we are *at present* acquainted. The first of these substances is secured in the form of powder to the end of a bougie by means of common glue, and before being applied to the obstruction, has a thin covering of bees-wax laid over it. The other is introduced into a hole, about the size of a pin's head, made in a common bougie, and covered with hog's lard. Either of these substances, when applied for a short time to the stricture, is dissolved, and they produce their effects by destroying it. In this way, the the membrane of the urethra is not so liable to be injured, as in the other modes of applying caustic substances; still, however, it must in many instances suffer; for when the caustic becomes liquefied, the disposition of all matter, and these in common with others, to run toward the orifice of the urethra, must greatly injure or entirely destroy the membrane, anterior to the stricture.

Even, however, where it has been beyond doubt ascertained, that permanent strictures exist, the use of internal medicines and external applications, during our attempts to remove them, are absolutely necessary. In all such strictures, even of a permanent nature, there is, according to the time they have existed, some degree of spasmodic action still remaining in them, which, in almost every instance, cannot suffer dilatation without these applications being previously made.

Any contrivance, however ingenious, for the application of caustic to strictures in the urethra, must be liable to one insurmountable objection. What I allude to is, the impossibility of preventing the caustic, liquefied by the moisture of the parts, from spreading over and destroying an extensive and healthy surface, particularly anterior to the obstruction. This, apply the caustic in what way we may, cannot be avoided; and this ought, with other very forcible reasons, to deter us from using it, unless where the most unequivocal marks of *permanent* stricture exist in the urethra.

The caustic, even before it can reach a stricture, must, from the mucus constantly secreted from the membrane of the urethra, be so completely diluted, or so entirely shielded, as to render it almost inert, *and unless assisted by considerable force*, must be, in many instances, incapable of acting on them with much vigour. And, even allowing it to be applied in the most favourable way, the difficulty of destroying the obstruction without such force, is greater than is commonly imagined. Under these circumstances, it labours under double the disadvantages even of the common bougie, when applied in the rudest manner; the force used in both is equally great, and, inert as the caustic may be rendered by dilution, which may prevent it from burning through a hardened and thickened stricture, it is still sufficiently active to injure, if not destroy, all, or most of the delicate membranes anterior to the strictured part.

Fistula in perinæo, I believe to be sometimes occasioned by obstructions in the urethra, either of a spasmodic or of a permanent nature; but I have no hesitation in asserting, the increased action brought upon the parts by the perseverance in the use of the caustic, or even the bougie, for a great length of time, has been a more frequent cause of this disease, than either of the above.

Mr. Carlisle inform us, in page 290 of the 3d volume of the Medical and Physical Journal, that a young gentleman, to whom he applied the caustic for a stricture, near the bulbous part of the urethra, had a hæmorrhage produced by it, which continued seven days; in the two first, he lost four pounds of blood, and nearly as much afterwards. He also has heard of some persons who have actually died of this kind of hæmorrhage.

"The young surgeon," this gentleman justly observes, "filled with the statements contained in books, and the cures so easily performed at the Lecture table, sets out in the profession without fearing the management of any recorded disease; he flatters the hopes of the patient, tries his skill, having, in his own mind, from the first, a full assurance of success; and too often meets with the bitterest disappointment." The truth of this picture, in the treatment of disease in general, we must all have felt, and from the smooth reasoning used by those, who are in the daily habit of using caustic bougies, and from the light and easy manner in which some attempt to treat the subject, the author has justly applied it to that particular practice.

When stricture in the urethra, either of a spasmodic or permanent nature, occasions a swelling of one or both testicles, this obstruction acts like a foreign substance in these parts; similarly to the use of too strong injections, which are very commonly the cause of the same complaint. The cause, however, being withdrawn or removed, the testicles resume their healthy action, which while it remains, no application to these glands themselves can effect. Although, therefore, this should be the case, it does not, as is too often done, entitle any one, on the appearance of swelled testicle, to thrust a bougie into the urethra, which can only cause spasm of these parts, which did not exist before, and enable him to term this permanent stricture, and then apply caustic for its removal. The properly regulated use of the bougie, even if spasm had previously existed, might have answered all useful purposes. I say the properly regulated use of the bougie—I do not mean its indiscriminate application; for, in this confused way of applying it, the swelling of the testicle, so so far from being removed is often increased, and is more frequently produced by it.

In this place I decline giving a number of cases. The aguments I have used, and the opinions I have formed, are here presented in a condensed view from the general range of those cases, &c. which have, for years past, come under my observation.

I shall now proceed to the consideration of Mr. Home's works on the subject of Stricture.

( To be continued. )

